# Estimating PQoS of Video Streaming on Wi-Fi Networks Using Machine Learning

**DOI:** 10.3390/s21020621

**Published:** 2021-01-17

**Authors:** Maghsoud Morshedi, Josef Noll

**Affiliations:** 1 EyeNetworks AS, 0680 Oslo, Norway; 2 Department of Technology Systems, University of Oslo, 2007 Kjeller, Norway; josef.noll@its.uio.no

**Keywords:** perceived quality of service (PQoS), machine learning, Wi-Fi, video streaming, QoE

## Abstract

Video on demand (VoD) services such as YouTube have generated considerable volumes of Internet traffic in homes and buildings in recent years. While Internet service providers deploy fiber and recent wireless technologies such as 802.11ax to support high bandwidth requirement, the best-effort nature of 802.11 networks and variable wireless medium conditions hinder users from experiencing maximum quality during video streaming. Hence, Internet service providers (ISPs) have an interest in monitoring the perceived quality of service (PQoS) in customer premises in order to avoid customer dissatisfaction and churn. Since existing approaches for estimating PQoS or quality of experience (QoE) requires external measurement of generic network performance parameters, this paper presents a novel approach to estimate the PQoS of video streaming using only 802.11 specific network performance parameters collected from wireless access points. This study produced datasets comprising 802.11n/ac/ax specific network performance parameters labelled with PQoS in the form of mean opinion scores (MOS) to train machine learning algorithms. As a result, we achieved as many as 93–99% classification accuracy in estimating PQoS by monitoring only 802.11 parameters on off-the-shelf Wi-Fi access points. Furthermore, the 802.11 parameters used in the machine learning model were analyzed to identify the cause of quality degradation detected on the Wi-Fi networks. Finally, ISPs can utilize the results of this study to provide predictable and measurable wireless quality by implementing non-intrusive monitoring of customers’ perceived quality. In addition, this approach reduces customers’ privacy concerns while reducing the operational cost of analytics for ISPs.

## 1. Introduction

Video on demand (VoD) services are becoming popular among Internet users such that video traffic will grow around 30% annually by 2025 [1]. YouTube has attracted approximately 44% of users among online streaming platforms such that there were an estimated 1.68 billion users of YouTube in 2019 [2]. On the other hand, recent Wi-Fi technologies such as 802.11ac/ax and fibre broadband have promised to provide high throughput connectivity, satisfying data-intensive applications such as video streaming. Users expect to stream online ultra-high definition videos on platforms such as YouTube uninterrupted everywhere in homes and buildings.

The 802.11 networks were initially designed with the best-effort mechanism in mind and QoS improvements were later added to the standard. Many factors affect the quality of wireless communication and 802.11 QoS amendments only address quality categories, while network issues are still annoying end-users. With increasing Wi-Fi penetration in homes, it is reported by Internet service providers (ISPs) that half of all technical-related service calls are due to problems with Wi-Fi networks [3]. The number of technical calls has increased in recent years because streaming VoD services are growing in popularity and customers consider ISPs to be responsible for any quality degradation.

Therefore, ISPs have been attempting to monitor the quality of services using external measurement tools, such as packet captures and speed tests. However, the packet capture approach imposes excessive cost and in some cases is not convenient. Speed tests only measure the maximum bandwidth of the Internet and do not capture wireless transient issues, so users experiencing wireless connectivity issues may remain frustrated. However, users are more concerned with the quality of applications rather than the maximum bandwidth, resulting in the need to measure users’ perceived quality of service (PQoS) rather than actual QoS. ISPs have been requesting user feedback through the application interface or user surveys in order to assess the quality of the user experience (QoE), which often represents users’ general opinions and is also influenced by non-technical factors such as price and social expectation. Hence, measuring PQoS has been preferred to eliminate the process of feedback campaigns and to assess the impact of technical factors on user satisfaction instead of the general opinion about a service.

The essence of the quality of users’ experience has motivated various research projects to present approaches for estimating the quality of experience on Wi-Fi networks. The majority of studies proposed methods to estimate the QoE, although they have only used network performance parameters for estimating service acceptability. For example, Ligata et al. used a subset of Wi-Fi network performance parameters and YouTube streaming parameters in order to predict QoE classes in the form of mean opinion score (MOS) values [4]. Likewise, Bhattacharyya et al. attempted to involve clients by measuring QoE and reporting to the software defined network (SDN) access point (AP) to apply queue prioritization for serving high-priority streaming requests in a network and consequently to improve the QoE by using reinforcement learning [5]. On the contrary, Morshedi presented a conceptual model to assess the service acceptability in the form of PQoS, which only uses network performance parameters [6]. Hence, it is desirable to distinguish between methods that use only technical factors and those leveraging user surveys to consider general opinion by using the term PQoS and QoE, respectively. To our knowledge, related works used external measurements to collect generic network performance parameters such as packet loss, throughput, delay and bandwidth on networks operating legacy 802.11g/n/ac standards to train machine learning algorithms. External measurements are often end-to-end and cannot distinguish problems between wired and wireless networks. Furthermore, all the network measurements, including raw data collected, were analyzed on the ISPs’ infrastructure, which raises privacy concerns. 

Therefore, this paper presents methods to estimate the PQoS of video streaming, in particular YouTube streaming using machine learning on the edge of the network. The 802.11 network performance parameters collected from off-the-shelf Wi-Fi access points along with YouTube streaming parameters were used to train machine learning algorithms to estimate the PQoS of YouTube video streaming. Thus, YouTube application parameters such as startup delay and rebuffering events were collected during YouTube streaming in order to classify the PQoS in the form of MOS values in the range of 1–5 (1 = bad, 2 = poor, 3 = fair, 4 = good, 5 = excellent). Meanwhile, 802.11a/g/n/ac/ax specific network performance parameters were collected from off-the-shelf Wi-Fi access points and labelled with MOS values in order to produce 4 datasets. Finally, supervised learning algorithms were trained in order to select the optimal machine learning technique.

As a result, the proposed approach could classify the PQoS of YouTube streaming correctly at least 93% of the time. Indeed, this study demonstrates that variation of the network performance parameters on the Wi-Fi access points highly correlates with the user’s perceived quality of YouTube streaming. Seven machine learning algorithms were chosen to be trained with datasets containing more than 230 802.11 network performance parameters. Among the machine learning candidates, this study indicated that the logistic model tree (LMT) performed best regarding accuracy, interpretability and computational cost criteria. Furthermore, network performance parameters were investigated to infer the cause of quality degradation in 802.11 networks. Finally, the machine-learning model as a script will be transferred to the access point in order to estimate PQoS on the network edge and to send the MOS value and the related parameters to the cloud infrastructure for further investigation.

This study intends to help ISPs and technical support teams to continuously monitor PQoS of video streaming in customer premises and even to identify the cause of quality degradation on the wireless network. Indeed, this study helps ISPs to perform proper proactive measures to deliver carrier-grade quality to customers and consequently reduce dissatisfaction and customer churn. This study will be extended to estimate the PQoS of other popular applications on Wi-Fi networks as well.

The rest of the paper is organized as follows: tools and methodology are described in Section 2. The results of data collection and training machine learning algorithms are presented in Section 3. Section 4 discusses the results and implications of the machine learning model and Section 5 presents related work for estimating QoE and PQoS using machine learning. Finally, Section 6 concludes the study and presents a future research roadmap.

## 2. Materials and Methods

The current study involved estimating the PQoS of YouTube streaming on Wi-Fi networks using machine learning techniques in homes and buildings. A series of experiments were performed to collect the 802.11 network performance parameters from Wi-Fi access points and labelled them with MOS values. Then, four datasets were produced for each of the 802.11g/n/ac/ax standards in the 2.4 GHz and 5 GHz frequency bands. Finally, seven machine learning algorithms were trained to find the best algorithm in terms of accuracy, interpretability and computational cost criteria. 

Both YouTube streaming and network performance parameters were collected in order to produce datasets for training and testing the machine learning algorithms. YouTube streaming parameters such as startup delay and rebuffering events were collected using the developer tools of web browsers playing YouTube high definition (HD) videos on mobile devices. Meanwhile, the 802.11 network performance parameters were collected from commodity Wi-Fi access points (APs). 

In order to collect YouTube video streaming parameters, JavaScript media events were used to capture events that can represent the quality of streaming. Among many media events, four were selected as representative of streaming parameters to calculate the MOS values. The first parameter is the startup delay, which indicates the time needed to fill the playing buffer before starting the playback. Viewers expect fast startup playback and will begin to abandon a video if the startup delay is longer than 2 s; for every additional startup delay in seconds, around 6% of the audience will abandon the video [7,8]. Since YouTube leverages the adaptive bit rate, the second parameter is the playback resolution, which indicates resolution changes during playback. Indeed, video resolution can significantly affect the startup delay or rebuffering, and can reduce the startup delay. The third and fourth parameters are the number of rebuffering events and the rebuffering duration. Rebuffering events that last only 1% of the total length of the video will reduce viewers’ engagement to continue watching the video by nearly 5% [7,8]. 

In experiments, wireless devices streamed City Walking Tours videos in 1080 p (pixel) as the reference resolution in YouTube in order to ascertain that the video frames were not static. During the playback, YouTube streaming parameters were collected every 3 s using a JavaScript script within the developer tools of the web browser. In the next step, the collected parameters should be represented as a mean opinion score (MOS) in which subjective assessment is the average of opinions or votes in the range of 1–5 (1 = bad, 2 = poor, 3 = fair, 4 = good, 5 = excellent), collected for a given condition [9]. There are different models to produce MOS values from video streaming performance parameters, which may not produce exactly the same MOS value. Mok et al. [10] presented a regression model that can produce MOS values according to the startup delay, the number of rebufferings and rebuffering duration parameters as follows:

(1) $$MOS=\;4.23\;-\;0.0672L_{ti}-\;0.742L_{fr}\;-\;0.106L_{tn}$$

In this model, *L_ti_*, *L_fr_*, and *L_tn_* are representative of the startup delay, the number of rebufferings and duration of rebuffering, respectively. While the abovementioned model produces MOS values, it does not consider the adaptive bit rate mechanism to avoid excessive rebuffering by downgrading the video streaming resolution. Therefore, this paper uses the findings of Asan et al. [11] to reflect the impact of switching from 1080 p as the reference condition to 480 p and 240 p in Equation (1) by reducing the overall MOS by 0.49 and 1.66 units, respectively. While Asan et al. [11] addressed only downgrading the resolution from 1080 p to 480 p and 240 p, the YouTube platform also implemented additional resolutions such as 720 p and 144 p. Thus, this study extended the findings of Asan et al. and calculated the impact of YouTube quality switches from 1080 p to 720 p and 144 p, as presented in Table 1.

As a result, our work leverages the following model in order to produce MOS values using YouTube streaming parameters on the Wi-Fi network, where *L_ti_*, *L_fr_*, *L_tn_*, and *R* are representative of startup delay, the number of rebufferings, the duration of rebuffering and the MOS impact of resolution downgrade, respectively.

(2) $$MOS\;=\;\;4.23\;-\;0.0672L_{ti}-\;0.742L_{fr}\;-\;0.106L_{tn}-\;R$$

The 802.11 network performance parameters were collected using a Bash script that collects parameters related to the following categories: 802.11 interface driver, multimedia access categories, connected clients’ counters for each frequency band in commodity Wi-Fi APs. In a typical Wi-Fi AP, the script collects more than 240 parameters for each frequency band interface and 48 parameters per client. The script collected all 802.11 counters on average at 1 s intervals and then sent the collected data over HTTP to an Elastic stack [12] platform as our cloud-based monitoring platform. The monitoring platform parses the received data to produce searchable fields and then indexes them in the ElasticSearch search engine for further analysis.

**Figure 1:**
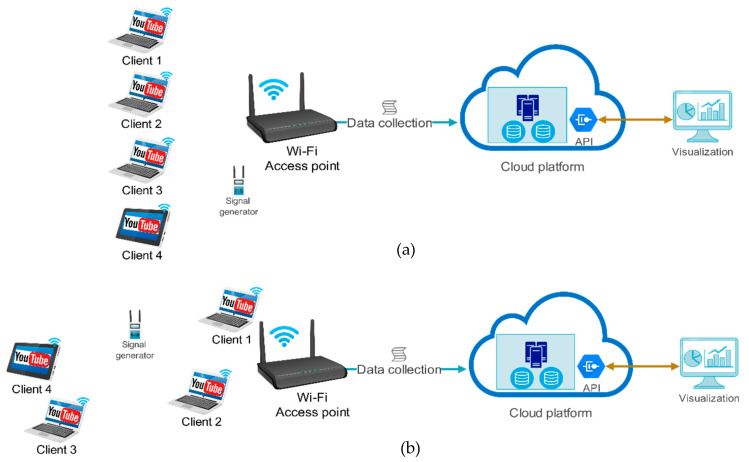
The experiment setup (**a**) when all clients were close to Wi-Fi AP and (**b**) when clients were placed in different rooms.

This study used off-the-shelf AirTies 4920 AP and Zyxel 5501 Internet gateway/AP in the experiments. Each of the APs has two wireless interfaces serving the 2.4 GHz and 5 GHz frequency bands. Therefore, four groups of experiments were conducted in order to investigate YouTube streaming in both the 2.4 GHz and 5 GHz frequency bands. The AirTies 4920 was used to test the 802.11n/ac standards (2.4 and 5 GHz) and Zyxel 5501 was used to test the 802.11ax standard (2.4 and 5 GHz).In each experiment, two different setups were implemented to connect four wireless devices to a Wi-Fi AP. Figure 1a depicts four wireless devices connected to an AP when they were close together and approximately 2 m from the AP in order to simulate a round-table scenario in a home environment. Figure 1b depicts four wireless devices located in different rooms in a home in order to simulate the most common scenario in home Wi-Fi. Three different laptops and a tablet were used to test the 802.11n and 802.11ac standards, while four Intel NUC with Intel Wi-Fi 6 AX200 adapters were used to test the 802.11ax standard in similar setups. Table 2 presents the characteristics of the wireless devices and their wireless network adapters, which were used in the experiments for streaming YouTube videos. At the beginning of each experiment, a data collection Bash script was started on the AP, and after a minute, the first device was started, streaming a YouTube video on the web browser. The wireless devices started streaming YouTube videos every 3 min one after another, and after the fourth device had streamed the video for 5 min, all the devices stopped streaming. Finally, the data collection Bash script on the AP was stopped after all four devices stopped streaming YouTube videos. 

**Figure 2:**
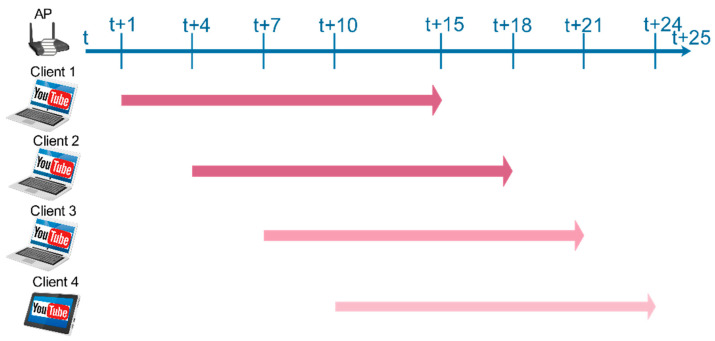
The timeline for streaming a YouTube video for each client during the experiments.

Figure 2 illustrates the timing of streaming YouTube videos in each client and when the Bash script starts and ends during the experiments. Meanwhile, the JavaScript script recorded the video streaming parameters for 14 min in each device. Furthermore, a signal generator was used to generate 802.11 packets and raw signals in order to simulate different kinds of interference in the communication environment during the experiments.

After data collection, the PQoS of YouTube streaming was represented in the form of MOS classes (1–5) using Equation (2). Thus, the 802.11 network performance parameters were labelled with the respective 1–5 MOS value in order to produce datasets for supervised learning. As a result, four datasets were produced, including 802.11n 2.4 GHz (2n), 802.11ax 2.4 GHz (2ax), 802.11ac 5 GHz (5ac) and 802.11ax 5 GHz (5ax). 

Machine learning techniques were leveraged to estimate the PQoS of YouTube streaming in the form of MOS values. This study established three criteria for selecting the most suitable machine learning algorithm among numerous existing algorithms. The first criterion was the maximum accuracy of the classifying instances, with a particular interest in classifying lower MOS values. The second criterion was interpretability such that the machine learning model can be easily understandable to facilitate tracing and inferring the cause of quality degradation on Wi-Fi networks. Miller presented a commonly cited definition of interpretability as how well a human can understand the logic of decisions made in the given domain and explain them to others later [13]. When the interpretability of a model is high, an observer can understand why a prediction has been made and which kind of decisions resulted in the prediction. The interpretability criterion has several advantages including allowing observers to extract the knowledge captured by the model, detecting biases in preferring specific group in the prediction, enhancing social acceptance and facilitating the debugging and auditing process. The third criterion was computational cost, such that the machine learning model can be implemented on edge devices capable of low-end computation.

This study leveraged Weka workbench [14] to train J48 (C4.5), Logistic model tree (LMT), reduced error pruning tree (REPTree), Naïve Bayes tree (NBTree), Ripple Down Rule learner (RIDOR) and multilayer perceptron (MLP) algorithms as representative of different categories of machine learning algorithms. The ZeroR algorithm was selected as the baseline algorithm to assess the efficiency of the candidate algorithms. Prior to the machine learning experiments, this study performed a resampling technique in order to find the optimal split between training and testing sets and therefore used 70% of instances for training and 30% for testing purposes.

At the end, the machine learning model will be transferred to the Wi-Fi access point as a script to take advantage of edge computing for estimating the PQoS of video streaming. As a result, the status of PQoS in form of MOS values along with parameters used to calculate the PQoS will be sent to the cloud platform for further investigation by support and operation teams.

## 3. Results

In this study, more than 16 experiments, as described in the previous section, were performed in order to investigate the PQoS of YouTube streaming on Wi-Fi networks. In the experiments, YouTube streaming and wireless network performance parameters were collected for training seven machine learning algorithms to estimate the PQoS of YouTube streaming using only wireless network performance parameters in homes and buildings. After performing the experiments, the wireless network performance parameters collected from a commodity Wi-Fi access point (AP) were labelled with the PQoS of YouTube streaming in the form of mean opinion score (MOS) classes in the range of 1–5 in order to produce datasets for training machine learning algorithms.

Equation (2), presented in the previous section, produced MOS floating-point values from the YouTube performance parameters (startup delay, number of rebufferings, duration of rebuffering and resolution switches). Notably, the model never produces an MOS value of 5, so good quality can be considered to be the optimal quality in this study. The floating-point values produced by the model were rounded to the nearest integer number to discretise the quality aligned with the MOS description in the range of values of 1–4 (1 = bad, 2 = poor, 3 = fair, and 4 = good). Furthermore, outputs of the model that were below one were considered as bad quality so they were adjusted to be MOS one in the datasets.

As a result of the experiments, four datasets were produced, namely 802.11n 2.4 GHz (2n), 802.11ax 2.4 GHz (2ax), 802.11ac 5 GHz (5ac) and 802.11ax 5 GHz (5ax). Table 3 presents the total number of instances, number of instances containing missing values, number of features and number of MOS labels in each dataset. As can be seen in Table 3, the majority of instances were labelled with MOS 4, where it was expected that the users experienced good quality. Besides, the number of missing values in 802.11ax was considerable because clients were often disconnected from the AP due to poor data channel conditions during the experiments and client-related 802.11 performance parameters were missing. The considerable number of instances and features in each dataset increased the computational complexity, so specific algorithms such as the multilayer perceptron (MLP) took hours for training with each dataset.

This study used the ZeroR algorithm as a baseline algorithm to assess the efficiency of the machine learning algorithms. For example, 74% of 802.11ac 5 GHz (5ac) instances were labelled as MOS 4, so the accuracy of the candidate algorithms should be at least 74%. In comparison to the ZeroR baseline, six other algorithms with the highest accuracy were selected from the decision trees, rule-based, functional algorithms, Bayesian and neural networks categories. The J48 (C4.5), Logistic model tree (LMT), reduced error pruning tree (REPTree), Naïve Bayes tree (NBTree), Ripple Down Rule learner (RIDOR) and multilayer perceptron (MLP) algorithms were selected for further analysis.

**Figure 3:**
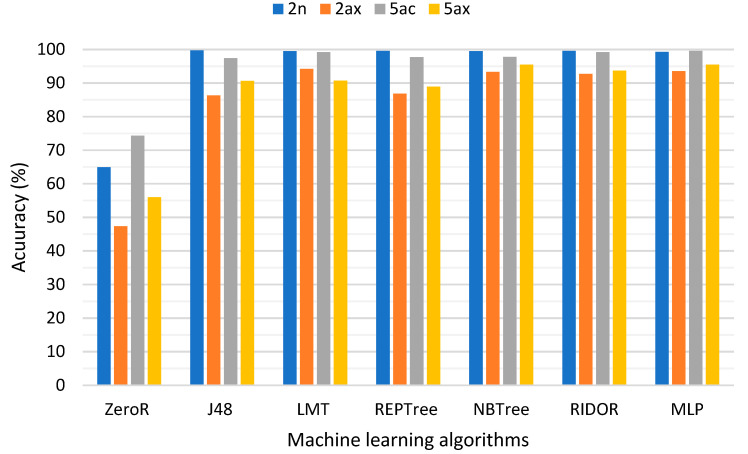
Percentage (%) of correctly classified labels by each of seven ML algorithms.

Figure 3 presents the accuracy percentage of the ML algorithms trained with the 4 datasets. The accuracy of ZeroR as the baseline algorithm was more than 47% in all datasets, which indicates that the majority of instances were labelled as good quality in the datasets. However, other algorithms performed substantially better than ZeroR such that their accuracy was more than 86% in all datasets. Since the accuracy of all seven algorithms in Figure 3 was quite similar, the interpretability and computational cost criteria were used for further comparison. 

The multilayer perceptron (MLP) achieved the highest accuracy, followed by the NBTree, RIDOR and LMT algorithms. However, the accuracy percentage cannot alone provide a precise assessment of how the ML algorithms performed in predicting MOS classes correctly. Therefore, a confusion matrix can help assess the amount of true and false positives for better comparison between ML algorithms. Table 4 presents confusion matrixes for the LMT models produced for the 2n, 2ax, 5ac and 5ax datasets. As can be seen in Table 4, the LMT models performed quite well in predicting poor PQoS levels by achieving the highest true positives for the MOS = 1 and MOS = 2 classes. The performance of the J48 (C4.5) and REPTree decision tree algorithms were quite similar and placed them at the bottom of the accuracy ranking. All seven algorithms trained with 802.11ax 2.4 GHz (2ax) failed to achieve as high accuracy as the other datasets, probably because 43% of instances contained missing values (see Table 3). Regardless of missing values, data augmentation techniques such as SMOTE can enhance the classification accuracy of ML algorithms such that applying SMOTE to 5ax dataset enhanced the accuracy to 97%.

The interpretability criterion can facilitate inferring of the cause of quality degradation on Wi-Fi networks. Therefore, models that are easy to interpret and trace are of particular interest to be selected for estimating the PQoS of YouTube streaming. The tree-like models produced by decision tree algorithms are easy to interpret, so J48, LMT, REPTree and NBTree can fulfil the interpretability criterion better than MLP and RIDOR. The tree-like models are preferred over other algorithms because the observer can explain the tree by decomposing the decision path into the steps per feature. Indeed, a decision tree can be tracked and explained by thresholds added to each decision node. The tree structure generally provides a simple visualization and it can capture interactions between features. In addition, a tree structure provides a contrastive explanation such that an observer can always compare the prediction with other options on the other leaf nodes. Not least, a tree structure presents the order of feature importance in the prediction model, e.g., the root node has the highest impact because it splits dataset into two major groups.

While LMT and NBTree produce a tree-like structure, they use logistic regression and Naïve Bayes functions, respectively, in their leaves. J48 (C4.5) and REPTree were the best ML algorithms with respect to the interpretability criterion. However, J48 and REPTree could not achieve high accuracy compared to the LMT and NBTree algorithms. Therefore, LMT and NBTree appear to be the best ML algorithms in terms of accuracy and interpretability. At the bottom of the interpretability ranking, MLP and RIDOR were placed, respectively.

**Figure 4:**
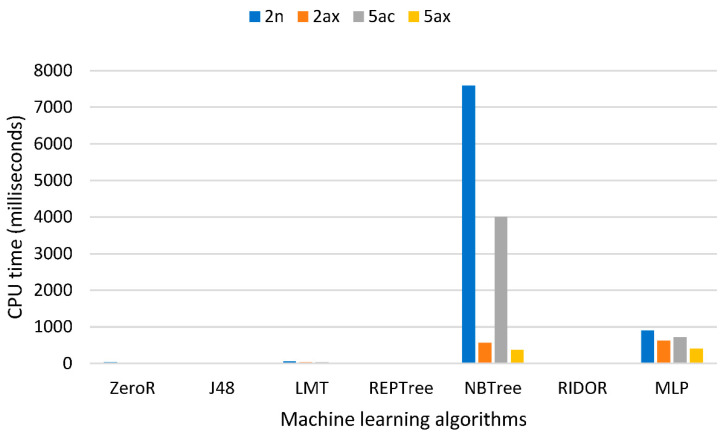
CPU time in milliseconds required for the testing phase of 30% of datasets.

The third criterion was the computational cost of implementing ML models, such that algorithms that consumed the least CPU time were preferred. Figure 4 presents the CPU time in milliseconds spent for testing 30% of instances in the datasets in a virtual machine with four Intel Haswell 2.6 GHz vCPU and 16 GB memory. As shown in Figure 4, J48, REPTree, RIDOR and LMT were the least CPU-demanding algorithms among the seven algorithms. The MLP and NBTree were CPU-demanding algorithms, took hours to be trained and were thus placed at the bottom of the computational cost ranking.

Table 5 presents the overall assessment of the ML algorithms trained in this study with respect to accuracy (A), interpretability (I) and computational cost (C) criteria. Generally, complex algorithms such as MLP achieves better accuracy and demands higher computation, which was treated as a black box when it comes to interpretability and explaining to decision-makers. Hence, there should be a trade-off between interpretability and accuracy. Since the accuracies of algorithms were quite similar, the interpretability and computational cost were determining factors in this comparison. The ultimate goal was to produce a model that can be executed on APs with low computational capabilities; meanwhile, operation teams can understand the prediction made by the model to identify the root causes of any quality degradation in wireless networks. As can be seen in Table 5, among the tree structure algorithms, the LMT and NBTree algorithms could predict the perceived quality accurately, while they present a more interpretable model than RIDOR and MLP algorithms. The J48 and REPTree had higher interpretability than LMT and NBTree, but they were not as accurate as LMT and NBTree predicting 2ax and 5ax wireless networks. Finally, the computational cost criterion was used as a tiebreaker between NBTree and LMT algorithms. Therefore, the LMT algorithm was selected as the best algorithm to produce a model to estimate the PQoS of YouTube streaming on Wi-Fi networks in homes and buildings.

The results demonstrated that the machine learning algorithms can correctly estimate the PQoS of YouTube streaming more than 90% of the time using only the 802.11 network performance parameters on different brands and standards of Wi-Fi APs in homes. While the datasets produced in this study may not represent all wireless medium conditions in Wi-Fi networks, the results demonstrated the applicability of the approach used in this study. Nevertheless, training machine learning algorithms in various wireless conditions can improve the precision of ML models over time.

## 4. Discussion

This study could classify the PQoS of YouTube streaming correctly more than 93% of the time using the 802.11 specific network performance parameters in homes and buildings. The results of this study indicate that 802.11 specific network performance parameters on Wi-Fi access points highly correlate with the perceived quality of video streaming and in particular YouTube streaming on Wi-Fi networks.

Given the analysis of the 802.11a/g/n/ac/ax standards, this study was able to achieve higher accuracy in predicting the quality of YouTube streaming compared to the results achieved in the literature [15,16,17,18,19]. While these related works used Wi-Fi as an access network for their experiments, they did not leverage 802.11 specific performance parameters for predicting the PQoS of YouTube streaming. However, this study produced multiple large datasets compared to the work in References [15,16,18,19] and was able to achieve higher accuracy regarding the majority class prediction as the baseline. Indeed, some machine learning approaches presented in Related Works could not achieve significant improvement compared to the majority class prediction baseline approach. Literature often leveraged external measurements to collect network performance parameters in order to train machine learning algorithms. Approaches presented in the literature increased the operational costs of analytics and raise customers’ privacy concerns while external measurements may not be convenient for customers or become expensive approach for ISPs. In contrary, this study presented a novel approach using machine learning and edge computing to address the abovementioned shortcomings of existing approaches in estimating the PQoS of video streaming. To our knowledge, this study is the pioneer in estimating the quality of wireless connectivity for Internet applications using only 802.11 specific network performance parameters on the off-the-shelf Wi-Fi access points.

This study leveraged YouTube streaming performance parameters comprising the startup delay, the number of rebufferings (stalling), duration of rebuffering and resolution (quality) switches in order to calculate the MOS value for each instance in the datasets. However, the related work used different approaches for predicting the quality of YouTube streaming, including binary-classes or three-classes prediction for each streaming parameter, binary (low and high) MOS prediction or using the ITU P.1203 methodology for calculating multi-class MOS values. Predicting the perceived quality of YouTube streaming using the individual prediction of streaming performance parameters could make the overall assessment challenging and would then require another round of prediction based on individual predictions. However, the binary classification may hide details required for granular analysis of quality deviations, and the machine learning algorithms could easily overfit the training data. Hence, representing the PQoS in the form of MOS values can facilitate the overall quality assessment process while providing a granular representation of perceived quality.

This study proposed the three criteria of accuracy, interpretability and computational cost for selecting the optimal machine-learning model capable of implementing on the low-end Wi-Fi access points in the form of edge computing. While the accuracy of the multilayer perceptron (MLP) and NBTree was the highest among the other candidates, this study selected LMT as the most suitable ML algorithm for estimating the PQoS of YouTube streaming. However, most of the related work only considered machine learning evaluation metrics such as the accuracy, precision, recall, F-measure, RMSE and ROC curve in order to select the best model for their problem domain. These machine learning evaluation metrics only consider the precision of the classification task, although a model with the best precision is not always applicable to the use case. Therefore, the applicability of the generated model should be considered as well during the model selection phase.

The LMT algorithm is a tree structure that benefits from logistic regression functions at its leaves using a stepwise fitting process and takes advantages of both tree and logistic regression. While J48 (C4.5) and REPTree were able to achieve the desired interpretability with the lowest computational cost, their accuracy was not suitable compared to the other four algorithms in this study. It is worth noting that meta-algorithms, such as Adaboost, can be used to enhance the classification accuracy of REPTree learner. However, MLP and NBTree achieved the highest accuracy, but imposed a considerable computational cost, while the rule-based RIDOR achieved the highest accuracy, but the rules were not as easy to interpret as the tree structure. Therefore, representing 802.11 network performance parameters in an LMT tree structure facilitated the identification of the key parameters affecting the PQoS of YouTube streaming on Wi-Fi networks.

**Figure 5:**
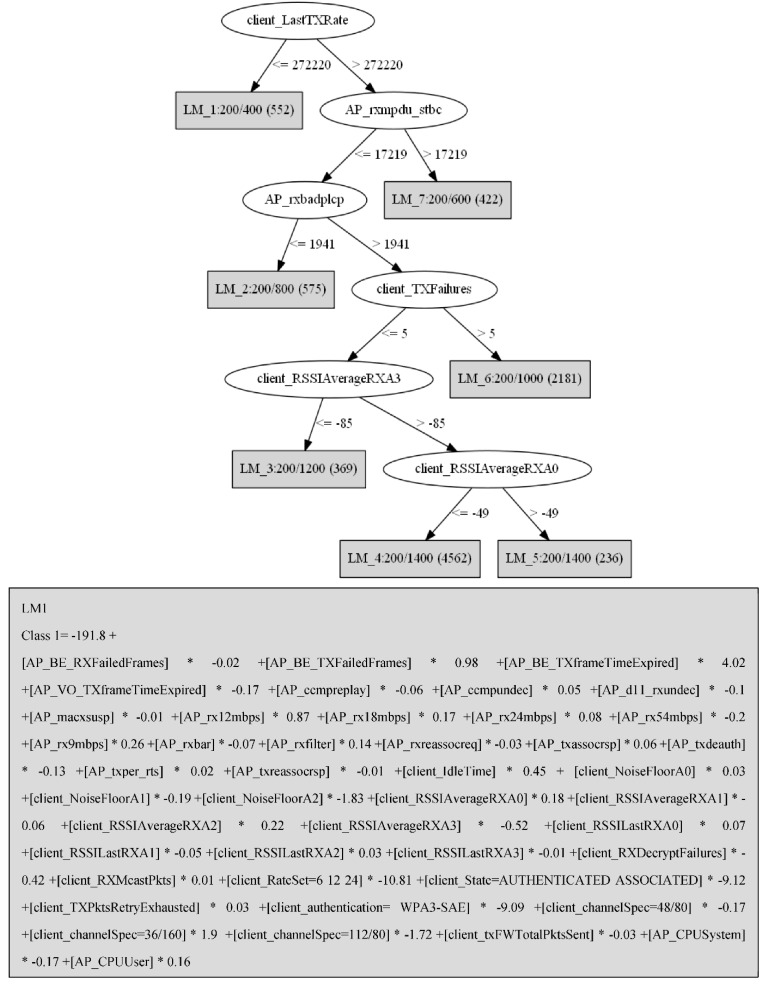
Output model for Logistic Model Tree (LMT) trained with 5ax dataset and logistic function for calculation of class 1 in LM1 as an example of logistic functions produced by the model.

As an example, the LMT tree trained with the 802.11ax 5 GHz (5ax) dataset will be investigated to showcase how the 802.11 performance parameters can indicate any changes in perceived quality while streaming YouTube videos on Wi-Fi networks. Figure 5 shows the tree model produced by the LMT algorithm trained with the 5ax dataset and features used in the logistic function of LM1 as an example for logistic functions produced by the model. The 5ax dataset consists of 299 features and six features had the highest importance so that they were used in the body of the LMT model, while the rest of the features were used in logistic functions to predict each MOS class. Features comprised 802.11 specific network performance parameters measuring, for example, signal strength, failed frames, association status, client-side signal strength and noise floor collected from the Wi-Fi AP operating at 802.11ax standard. While the J48 and REPTree are more interpretable, the LMT model was preferred because it achieved higher accuracy with a reasonably low computational cost. However, if the maximum interpretability had the highest priority, then J48 would be the best model in this use case. Hence, a trade-off between interpretability and accuracy was the basis to choose the LMT as the best algorithm in this study. 

As can be seen, the rate of the last frame transmitted to the client is selected as the root of the tree with a threshold of 272 Mbps, which has a logistic model function for each class as the left child. From 299 features in the 802.11ax 5 GHz dataset, six features were used in the body of the LMT tree and the remainder were used in the logistic model functions in the leaves. There are four logistic functions in each logistic model leaf, such that a function that results in a positive value determines the predicted class in that node. For example, if the logistic function of class 1 in the LM1 node results in a positive value, LM1 returns MOS = 1 as the predicted class. The rest of the features used in the body of the LMT tree provide the following impact on the quality of YouTube streaming on Wi-Fi networks.

**Figure 6:**
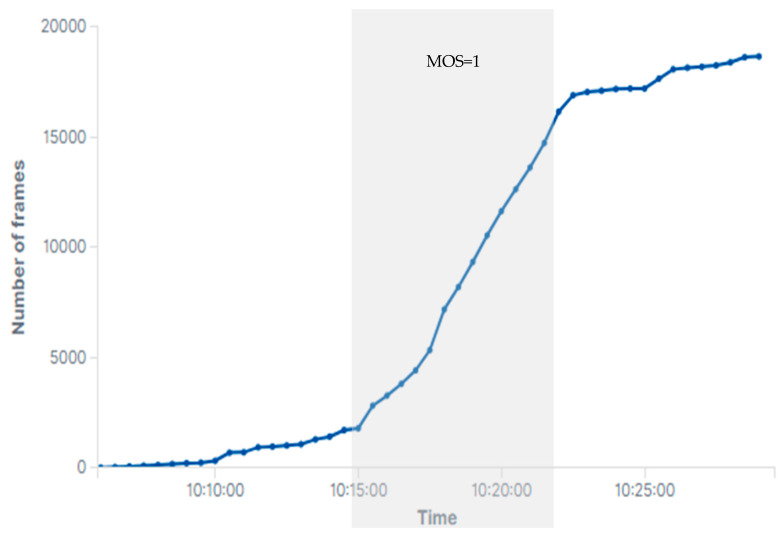
Number of received MPDU that used STBC in order to increase reliability.

The rxmpdu_stbc counter increments for each MAC protocol data unit (MPDU) received using a space-time block coding (STBC) technique. The STBC is used to transmit multiple copies of a data stream over multiple antennas in multiple-input multiple-output (MIMO) systems in order to increase the reliability of data transfer in harsh environments. The STBC technique is often used when the data channel is too poor to support one stream per antenna due to high scattering, reflection and refraction, or when it is expected to reach a longer range in beamforming [20]. The STBC technique can also increase the reliability of data transfer during channel fading. Channel fading can affect the throughput and received signal strength [21] and can therefore implicitly be identified by monitoring sharp fluctuations in the data rate of the last frame (client_LastTXRate) and the RSSI of the last frame per antenna (client_RSSILastRX) for each client. Therefore, a sharp increase in the number of rxmpdu_stbc can represent a poor channel condition and at least a 50% reduction in transmission throughput. Figure 6 illustrates a sharp increase in the number of received MPDU with STBC enabled, which represents a degraded PQoS with MOS = 1 during the experiments.

The rxbadplcp counter increments when the parity check of the physical layer convergence procedure (PLCP) header failed. The PLCP header contains the transmission rate, frame length and a single bit for parity error checking. Due to a weak error-checking mechanism, PLCP header corruption can impose packet loss and lower throughput rates. The main causes of PLCP header corruption are interference and a noisy channel condition.

The client-side transmit failures (txFailures) represent conditions in which frames are discarded because the number of retransmissions has been exceeded due to either short or long retry limits. A high number of failed transmissions can indicate whether a poor channel condition was temporary or long-lasting. However, a comparison between received retransmissions and failed transmissions can indicate whether the end-to-end data channel condition is poor or if one side only is experiencing a poor channel condition.

The received signal strength indicator (RSSI) is one of the most commonly used parameters in the quality assessment of wireless communication. While the RSSI has been commonly used, the IEEE has not standardized the correlation between the RSSI and RSS, so various devices report different RSSI values. Hence, it is recommended to consider the noise level or sensitivity to calculate the signal-to-noise ratio (SNR) for a more reliable comparison. This study used different devices during the experiments in order to reduce the probability of overfitting the RSSI to a product of a specific vendor. Here, the clients and AP contained 2×2 and 4×4 MIMO, respectively, so in the case of beamforming, it was expected to see different RSSI thresholds in the LMT model for antennas 1 and 4 on the AP.

This study was able to achieve promising results in estimating the PQoS of YouTube streaming on Wi-Fi networks using 802.11 specific performance parameters. The generated ML model will be executed on Wi-Fi APs to monitor the quality of wireless connectivity at customer premises while our approach avoids sending raw data to service providers’ infrastructure. In this form of edge computing, the PQoS will be calculated on Wi-Fi APs and if any quality degradation occurs the level of PQoS and 802.11 performance parameters that captured quality degradation will be sent to the ISPs’ infrastructure for further investigation by support and operation teams. This approach has three advantages: (i) preserving privacy by not sending raw data out of the customer’s premises, (ii) eliminating raw data processing in the service provider’s infrastructure and therefore reducing the operational cost of analytics for service providers, and (iii) providing a zero-touch tool for evaluating the perceived quality of wireless connectivity in homes and buildings.

## 5. Related Work

Various research articles have investigated the prediction of the perceived quality of YouTube streaming as the quality of experience (QoE) using machine-learning, while some of them have considered Wi-Fi networks as the access network in their experiment setups. Morshedi and Noll surveyed the literature on the prediction of PQoS using machine-learning on Wi-Fi networks and presented that most of the literature used generic network performance parameters for estimating PQoS using external measurements such as packet capture or ping tests [22]. While these research efforts have used different network performance parameters to predict the perceived quality of YouTube streaming, they have leveraged very similar YouTube application parameters, such as the startup delay, number of rebufferings (stalling), and resolution (quality) switches to calculate the MOS or label instances with individual parameters in the ML dataset.

Seufert et al. analyzed the stalling status of YouTube encrypted traffic using a random forest ML algorithm on Wi-Fi networks. They collected network performance parameters such as the download ratio, RX/TX bytes, RX/TX packets, and time-based parameters such as the delay for the first packet and the time between the first and last packet. They streamed 4714 YouTube video sessions for 180 s using a Selenium browser and JavaScript script recorded playback parameters such as the player state and resolution every 250 milliseconds. Videos streamed over Wi-Fi and LTE mobile networks with a maximum 20 Mbps bandwidth and traffic were captured using Tshark. The dataset comprised 4849 instances and more than 80% of these experienced no stalling, so they resampled the dataset in order to balance the instances with stalling and no-stalling labels and then trained a random forest algorithm with 80% of the dataset. As a result, they achieved a maximum accuracy of around 95% in predicting stalling in YouTube streaming [15]. Cárdenas-Angelat et al. investigated the prediction of QoE of YouTube streaming using deep learning on Wi-Fi networks. They attempted to predict the startup delay, resolution and stalling events using the amount of data sent and transmitted from smartphones using neural networks. The dataset comprised 1303 instances and they trained neural networks with 80% of the dataset. They were able to achieve around 90% accuracy in predicting binary classes of YouTube streaming performance parameters [16]. In other research, Bartolec et al. analyzed YouTube performance using ML on Wi-Fi networks and collected the startup delay, stalling, resolution and average bitrate using YouTube stats-for-nerds on two mobile phones and emulated the LTE network condition using the IMUNES tool. The dataset comprised 608 videos, but stalling occurred on only 20 videos, so stalling, trio-class resolution and startup delay of less than 3 s were omitted from the ML training phase. They achieved a maximum 95% accuracy in predicting the binary class of the average bitrate, 78% startup delay and 84% binary class resolution prediction using 10-fold cross-validation [17].

Selma et al. analyzed the perceived quality of YouTube encrypted streaming using packet captures and ML. They used the dataset produced by Reference [18] comprising 226 features extracted from TCPDump packet captures and the startup delay, number of stalls and video resolution in the form of binary labels. The YouTube videos were streamed using a Selenium browser on a PC and YouTube streaming parameters collected using the YouTubes Iframe API, while network conditions were emulated using Netem and TC tools. The dataset comprised 5488 instances for HTTPS and 5375 instances for QUIC streaming, of which 72% of the instances in the HTTPS dataset experienced startup delay of less than 10 s. They trained the Naïve Bayes, MLP algorithm, Decision Table, J48, LMT and random forest, AdaBoost and Bagging algorithms. They achieved a maximum accuracy of 80% with random forest for HTTPS startup delay with a 10 s threshold dataset. While 86% of 135,830 instances in the HTTPS stalling dataset experienced no rebuffering, they achieved 91% accuracy using the random forest as the highest accuracy among all datasets [19]. Likewise, Khokhar et al. investigated the prediction of the perceived quality of YouTube streaming using QoS parameters and they used the startup delay, number of stalls, quality switches and QoS parameters comprising the bandwidth, round trip time (RTT) packet loss rate and jitter. Their dataset comprised 48 features and only 12% of instances suffered from stalling, while only 7% of instances experienced timeout and 73% of instances experienced at least one quality switch. They used the ITU P.1203 recommendation to produce MOS classes and they achieved a maximum accuracy of 90% on all datasets using linear regression and random forest algorithms [23].

Wasserman et al. analyzed the perceived quality of YouTube streaming in cellular networks through smartphone measurements. They used the YoMo mobile application to collect the startup delay, resolution switches and the number of stalls along with cellular network performance parameters every second and user feedback after playback. They considered the binary classification problem in the form of two classes, namely better or worse than MOS = 4. They trained a single decision tree, SVM, KNN, Naïve Bayes and ensemble learning techniques such as AdaBoost and gradient boosting algorithms and were able to achieve an overall accuracy of around 90% on 10-fold cross-validation [24].

The review of the literature presented here indicated the need for more efficient approach than external measurements of network performance parameters because external measurements in customers’ premises may not be convenient and will increase the operational cost of customer support for ISPs. In addition, the literature often used small datasets produced by testing legacy Wi-Fi networks, which cannot represent wireless networks operating recent 802.11ax standards in the real world. Indeed, machine-learning models trained by small datasets can easily overfit or underfit the dataset and may not extend to unseen conditions in real-world use cases. Literature often presented approaches that collect all the measurement data to the service providers’ infrastructure which raise privacy concerns about data access, storage and analytics other than service operation purposes. Therefore, this study proposes a novel approach to use 802.11 specific network performance parameters, edge computing and machine-learning techniques to address abovementioned shortcomings.

## 6. Conclusions

While Internet service providers deliver high bandwidth using fiber deployments, the best-effort nature of 802.11 networks may prevent users from experiencing the maximum quality during video streaming. Research efforts have introduced various approaches to estimate the perceived quality of video streaming using machine learning. Existing approaches often leveraged external measurements to collect generic network performance parameters such as delay, jitter and packet loss to estimate the perceived quality of video streaming. However, external measurements may not be convenient or can impose an excessive cost for ISPs. In addition, existing approaches collect all measurements and perform analytics on the service providers’ infrastructure which increase operational costs and also raise privacy concern. Therefore, this paper proposed a novel approach for estimating the PQoS of video streaming, in particular YouTube streaming using only 802.11 specific network performance parameters on Wi-Fi networks. Hence, more than 16 streaming experiments were performed on Wi-Fi networks operating the 802.11g/n/ac/ax standards over the 2.4 GHz and 5 GHz frequency bands. Then, various ML algorithms were trained using 70% of the 2n, 2ax, 5ac and 5ax datasets and achieved 93–99% accuracy estimating the PQoS classes. As a result, the logistic model tree (LMT) was selected as the most suitable algorithm to estimate the PQoS of video streaming in terms of accuracy, interpretability and computational cost criteria. The generated ML model will be transferred to the Wi-Fi access point as a lightweight script to continuously monitor the PQoS of video streaming on Wi-Fi networks. The script will calculate the PQoS in the form of MOS values and report it to the service providers’ customer support. If the quality degrades, then it sends the 802.11 network performance parameters that captured quality degradation to the support and operation teams for further investigation. ISPs can utilize the results of this study to leverage machine-learning techniques to monitor the PQoS of video streaming in access network rather than the cloud environment. Correspondingly, ISPs can deliver predictable and measurable wireless quality for Internet connectivity and new upcoming services to their customers. In addition, our novel approach will reduce privacy concerns and operational costs of data analytics for ISPs.

While this study attempted to consider real-world patterns of video streaming in homes and buildings, it was not possible to capture all wireless conditions in the training datasets. Therefore, future studies can collect data from different environments in order to improve the precision of the machine-learning model. However, this study only focused on the video streaming application, while users utilize various types of applications such as video/audio conference or web browsing services. Hence, the authors will consider video conference and web browsing services in future studies.

## Figures and Tables

**Table 1:** Impact of YouTube resolution switches in overall mean opinion score (MOS).

Reference Resolution (Pixel)	Target Resolution (Pixel)	MOS Impact
1080	720	0.21 [This work]
1080	480	0.49 [Asan et al.]
1080	240	1.66 [Asan et al.]
1080	144	2.07 [This work]

**Table 2:** Characteristics of devices were used for experiment in this study.

Device Model	Network Adapter	Wireless Technology	Number of Devices
Dell Latitude 5480	Intel 8265	802.11a/n/ac	1
HP Elitebook 8460 P	Intel Centrino 6205	802.11b/g/n	1
Lenovo E531	Realtek 8812BU	802.11a/n/ac	1
Microsoft surface	Marvell AVASTAR Wireless-AC	802.11a/n/ac	1
Intel NUC6CAYH	Intel AX200	802.11ax	4

**Table 3:** Characteristics of datasets produced in this study.

Dataset	#Instances	#Missing Values	#Features	#MOS = 1	#MOS = 2	#MOS = 3	#MOS = 4
2n	13,039	96	240	681	542	3363	8453
2ax	12,762	5467	294	4728	593	1404	6037
5ac	7896	393	246	863	445	721	5867
5ax	8897	2285	299	2833	816	264	4984

# denotes Number of instances.

**Table 4:** Confusion matrix of LMT models produced for 2n, 2ax, 5ac and 5ax datasets.

	**2n Classified as**					**2ax Classified as**			
	**a**	**b**	**c**	**d**		**a**	**b**	**c**	**d**
a = 1	186	4	0	0	a = 1	1328	55	5	9
b = 2	2	170	2	0	b = 2	51	122	0	7
c = 3	0	1	978	2	c = 3	6	1	388	37
d = 4	0	1	6	2560	d = 4	13	5	32	1770
	**5ac Classified as**					**5ax Classified as**			
	**a**	**b**	**c**	**d**		**a**	**b**	**c**	**d**
a = 1	250	0	0	1	a = 1	803	0	0	74
b = 2	0	137	0	1	b = 2	18	207	0	3
c = 3	0	0	228	0	c = 3	6	1	64	3
d = 4	1	4	3	1744	d = 4	116	4	8	1362

**Table 5:** Overall assessment of ML algorithms with respect to accuracy (A), interpretability (I) and computational cost (C) criteria.

Dataset	J48			LMT			REPTree			NBTree			RIDOR			MLP		
	A	I	C	A	I	C	A	I	C	A	I	C	A	I	C	A	I	C
*2n*	+	+	+	+	+	+	+	+	+	+	+	−	+	−	+	+	−	−
*2ax*	−	+	+	+	+	+	−	+	+	+	+	−	+	−	+	+	−	−
*5ac*	+	+	+	+	+	+	−	+	+	+	+	−	+	−	+	+	−	−
*5ax*	+	+	+	+	+	+	+	+	+	+	+	−	+	−	+	+	−	−

## Data Availability

The datasets used to support the findings of this study are available from the corresponding author upon request.
